# Human P301L tau expression in mice alters neuronal excitability independent of neurodegeneration

**DOI:** 10.3389/fnins.2026.1814637

**Published:** 2026-09-04

**Authors:** Katie L. Dunton, Hugo Monchal, Brie E. Achorn, Betty Mandé-Niedergang, Corinne Roucard, Yann Roche, Alexis Evrard, Frank S. Menniti

**Affiliations:** 1 MindImmune Therapeutics, Inc., Kingston, RI, United States; 2 SynapCell SAS, Saint-Ismier, France; 3 Interdisciplinary Neuroscience Program, University of Rhode Island, Kingston, RI, United States; 4 George & Anne Ryan Institute for Neuroscience, University of Rhode Island, Kingston, RI, United States

**Keywords:** electroencephalography, neurodegeneration, neuroinflammation, tau, tauopathy

## Abstract

Variation in the microtubule-binding protein tau is causally implicated in numerous neurodegenerative diseases, including Alzheimer’s disease. However, the mechanisms by which such variation results in disease are not well understood. The JNPL3(P301L) mouse model mimics the effects of the P301L mutation on human tau associated with frontotemporal dementia. As previously reported, phospho-tau aggregates immunolabeled with AT8 increase with age in a rostral progression from the brainstem; however, there is relatively limited forebrain pathology by the time these animals die prematurely at approximately 1 year of age. In this study, we investigated the functional effects of P301L tau expression on electrophysiological signatures as a function of age in mice expressing P301L tau from 3 to 10 months. The P301L mice had a distinct electrophysiological phenotype of increased power in higher EEG frequency bands detected with a depth electrode in the hippocampus and, to a lesser extent, with dural surface electrodes over the frontal and parietal cortices. Significantly, this electrophysiological phenotype was present at the first recording at 3 months of age and did not differ over monthly recordings up to 9 months of age, after which point animals became moribund and were euthanized. P301L mice at 10 months of age also showed electrical synchronization to a 40 Hz tone (ASSR) that was not observed in WT mice and an increase in inter-trial coherence compared with WT mice. We confirmed that AT8, as well as markers of neuroinflammation, increased progressively with age from the hindbrain to the forebrain, as previously reported. Together, these data suggest an increase in neuronal excitability in P301L mice compared with WT, but that this phenotype is not correlated with the age-dependent accumulation of AT8-labeled tau aggregates and the accompanying neuroinflammation. We hypothesize that the expression of P301L mutant tau disrupted the physiological functions of tau, resulting in the functional electrophysiological phenotype. Whether this functional effect drives neurodegeneration or is a separable phenomenon has implications for understanding the mechanisms underlying the role of tau variation in neurodegenerative conditions.

## 1 Introduction

Tau is a family of proteins among whose physiological functions are the regulation of microtubule stability in axons throughout the central nervous system (Wang and Mandelkow, 2016; Arendt et al., 2016; Parra Bravo et al., 2024). Tau is encoded by a single gene, *MAPT*, which is expressed as multiple splice variants. In humans, splice variants encoded by exon 10 yield proteins that contain 3 or 4 microtubule-binding domain repeats (Andreadis et al., 1992). Human tau proteins are also expressed with one or two N-terminal inserts, thus yielding a total of six variants. Mouse tau is expressed with 3 or 4 microtubule-binding domain repeats but lacks N-terminal inserts (Lee et al., 1988). The interaction between tau and microtubules is dynamically regulated by phosphorylation at multiple sites on the protein (Stoothoff and Johnson, 2005).

Dysregulation of tau and tau phosphorylation is implicated in neurodegenerative disease (Spillantini and Goedert, 2013; Ballatore et al., 2007; Zhang et al., 2022). More than 100 mutations in *MAPT* have been identified that are either causal or increase the risk for several primary ‘tauopathies,’ including frontotemporal dementia, ALS, progressive supranuclear palsy, corticobasal syndrome, and chronic traumatic encephalopathy (Langerscheidt et al., 2024). Alzheimer’s disease (AD) may also be considered a tauopathy. In AD, intraneuronal aggregates of hyperphosphorylated tau are the major component of neurofibrillary tangles (NFTs), which are one of the two pathological hallmarks of the disease (Kowall and Kosik, 1987). Furthermore, the spatial and temporal progression of NFT accumulation from the temporal to the frontal cortex closely tracks the progression of disease symptoms and neurodegeneration (Braak and Braak, 1995; Braak et al., 2006). These associations have strongly supported the hypothesis that tau hyperphosphorylation and aggregation are causative factors in AD. However, there is no genetic linkage to AD for variation in *MAPT* or tau regulatory elements. This contrasts with the genetic linkage for mutations in *APP* and APP-metabolizing enzymes that result in altered production of β-amyloid that accumulates in extracellular amyloid plaques, the other pathological hallmark of AD (Citron et al., 1992; Mullan et al., 1992). The reasons why *MAPT* mutations can result in distinct neurodegenerative tauopathies, yet no tau variant is linked to AD, remain fundamental puzzles and a key area of research for the development of therapeutics for these diseases.

A variety of mouse genetic models have been created to investigate tau pathophysiology, largely focusing on how tau dysfunction damages neurons (Sahara and Yanai, 2023; Götz et al., 2007; Langness et al., 2025). Consequently, model development has skewed toward high levels of tau expression and other ancillary manipulations that result in robust neurodegenerative phenotypes that develop rapidly. However, AD and the tauopathies are insidious, developing over years- or decades-long prodromal phases (Benatar et al., 2024; Dubois et al., 2016; Benussi et al., 2022). Furthermore, these diseases develop in the absence of massive tau overexpression. This suggests that dysfunctional tau may have an impact on neuronal function during the prodrome that contributes to, or causes, neurodegeneration present at diagnosis and subsequent clinical progression. Thus, it is important to investigate the subtle effects of tau dysfunction that are prodromal to neuronal damage. This aspect of tau pathophysiology may be better studied in less aggressive tauopathy mouse models.

In this study, we investigated the effects of human tau expression in mice harboring the P301L substitution on neuronal function using quantitative EEG (qEEG) in JNPL3 (P301L) mice. This mouse line, developed by the Hutton lab (Lewis et al., 2000) and maintained at Taconic Biosciences, Inc., Rensselaer, NY, USA, was among the first tauopathy models but was superseded as more aggressive tauopathy models became available (e.g., Tg4510 mice) (Santacruz et al., 2005). JNPL3 mice carry a transgene encoding human tau that contains the P301L substitution associated with frontotemporal dementia. The mutation is harbored in a four microtubule-binding repeat domain/no N-terminal insert tau isoform. Expression is constitutive, driven by the mouse prion promoter (Borchelt et al., 1996), and levels of htau^P301L^ are reported to be approximately 2-fold higher than endogenous mouse tau. *In situ* hybridization revealed that transgene expression in these mice is largely neuronal, with strongest expression in the cerebellum and hippocampus. These mice develop tau deposits that increase with age and progress rostrally from the spinal cord and brainstem. We were interested in identifying *in vivo* functional consequences that emerge as a result of tau deposition that may be used as outcome endpoints in the testing of experimental therapeutics. Specifically, we investigated electrophysiological phenotypes measured as changes in the electroencephalogram (EEG) with age. We hypothesized that distinct EEG phenotypes would emerge progressively with age in parallel with the emergence of tau deposition. We found evidence for increased neuronal excitability in the frontal cortex and hippocampus. However, in contradiction to our hypothesis, this phenotype was present from 3 months of age and was stable through 10 months of age, the lifespan of these animals. Thus, the functional phenotype did not correlate with the progressive NFT accumulation reported previously. We conclude that the electrophysiological phenotype was the result of htau^P301L^ expression causing neuronal dysfunction but not neuronal degeneration.

## 2 Methods

### 2.1 Animals

Female P301L (STOCKTg[Prnp-MAPT*P301L]JNPL3Hlmc) mice and female WT control mice (STOCK [SW and B6D2F1]) were purchased from Taconic Biosciences Inc. (Rensselaer, NY, USA). Female P301L mice have more extensive tau pathology and develop pathology earlier in life than males (Sahara et al., 2014; Sahara et al., 2002). Thus, only female mice were used in these studies to reduce variability and the number of animals used. We did not adjust our experimental design to account for the hormonal cycle. Electrophysiological recordings were performed using mice housed at SynapCell (Bâtiment SYNERGY, ZAC ISIPARC, 38330 Saint Ismier, France). Studies were performed following the local Standard Operating Procedures of SynapCell, were approved by the ethical committee of the Grenoble Institute of Neurosciences, University Grenoble Alpes, and were performed in accordance with the European Council Directive of 22 September 2010 (2010/63/EU). Immunohistochemical analyses were performed on mice housed at the University of Rhode Island (Kingston, RI 02881, USA). The University of Rhode Island Institutional Animal Care and Use Committee reviewed and approved all studies. At both institutions, animals were maintained under standard laboratory conditions, with artificial light on a 12 h light/dark cycle with food and water available ad libitum. In all cases, efforts were made to minimize animal suffering and reduce the number of animals used. The P301L mice began to display a hunched posture and gait deficits from approximately 11 months of age, as previously reported (Lewis et al., 2000), and the experiments were terminated at this time.

### 2.2 Quantitative electroencephalography (qEEG)

The method used was similar to that used by our group (Serrats et al., 2025). Twelve P301L and 12 WT mice were used for electrophysiological recordings. Experimenters were blind to the genotype of the two groups. Animals were 2.5 months of age (± 1 week) at the time of electrode implantation. Stereotaxic implantation of the electrodes was performed under general anesthesia (isoflurane 2% in oxygen). Animals also received a subcutaneous injection of the analgesic buprenorphine (0.05 mg/kg) within 30 min before anesthesia. Animals were placed within the ear bars in a stereotaxic frame. Body temperature was monitored and maintained at a constant temperature during surgery. An ophthalmic gel was placed on the eyes to avoid drying of the cornea. The depth of anesthesia was verified before starting the surgery, and the breathing rate and cardiac rhythm were monitored visually during the surgery. The scalp was shaved and disinfected using povidone. The skin was incised, the skull was cleaned, and electrodes were implanted through the skull. Screw electrodes were placed in contact with the surface of the dura mater. Electrodes were implanted at stereotaxic coordinates (in mm) from the origin at Bregma (AP: anteroposterior distance; ML: mediolateral distance) and at dorsoventral depth (DV) from the surface of the skull (Paxinos and Franklin, 2019). One stainless steel screw (E363/96/1.6/SPC ELEC W/00-96×1.6MM (1/16) SCREW, PlasticOne) was placed over the left [AP 0.5, ML − 2, DV brain surface], right [AP 1, ML 0.5, DV brain surface] frontal cortex, and left parietal cortex [AP -3.2, ML − 2.5, DV brain surface]. A depth electrode (E363/2/SPC ELEC 0.008/.2MM SS, PlasticOne) was implanted in the right hippocampus [AP-2.4, ML 1.5, DV -2]. Finally, a reference stainless-steel screw electrode (E363/96/1.6/SPC ELEC W/00-96×1.6MM (1/16) SCREW, PlasticOne) was placed above the cerebellum (no precise coordinate was used for this reference electrode). All electrodes were connected to a female connector placed on top of the skull and secured in place using dental cement. After surgery and termination of anesthesia, the animals were monitored until fully awake before being returned to their home cage. The animals received another dose of buprenorphine at a maximum of 8 h after the initial dose. After electrode implantation, the mice were housed individually to prevent cage mates from damaging the electrodes.

After 1 week of recovery, electrophysiological signal integrity was assessed, and eight animals per group were chosen for further study. Experience at SynapCell in performing mouse EEG studies provided confidence that this number of animals per group would be sufficient to detect an age-related change in the EEG structure. Electrophysiological recordings were conducted once per month beginning at the age of 3 months through 9 months. Electrophysiological recordings were performed during the light phase on freely moving animals. Animals were placed in a plastic enclosure (17×17cm base size) and connected to the recording system via the head connector. Following 30 min of acclimatization in the recording chambers, electrophysiological signals were recorded for 120 min without interruption or disruption. Electroencephalographic (EEG) signals from cortical electrodes and local field potentials (LFP) from hippocampal electrodes were collected via the same interface. Signals were amplified and digitized using a CED 1401 interface before being acquired at 512 Hz using Spike 2 software (Cambridge Electronic Design Limited).

Raw EEG and LFP files were analyzed using MATLAB (MathWorks, Natick, MA, USA). The spectral power density (between 1 and 140 Hz) was calculated over 30-min periods using a fast Fourier transform (FFT). The spectra were then divided into classical frequency bands, and the total power in each band was computed. These were defined as: Delta (1–4 Hz), Theta (4–8 Hz), Alpha (8–12 Hz), Beta (12–30 Hz), Low Gamma (30–50 Hz), High Gamma (50–90 Hz), and Epsilon (90–140 Hz). The average spectral power within a band was calculated over the 120-min period of spontaneous EEG recordings to provide a single data point per recording day. The readings were the total power between 1 and 140 Hz and the absolute power of each spectral band at each electrode. Prism v10 (GraphPad) was used for statistical analyses. Statistical analysis of the differences in electrophysiological power in post-hoc analyses was performed using Dunnett’s test. The significance level was set at *p* < 0.05.

### 2.3 Auditory steady-state response (ASSR)

ASSR was investigated on the same animals used for qEEG, at 9–10 months of age in a session after the last qEEG recording. Electrophysiological signals were acquired using the same CED-based system described above for qEEG recordings. Following acclimatization in the recording chambers, electrophysiological signals were recorded for 10 min with no auditory stimulation. The subjects were then exposed to 360 repetitions of a train of sound pulses made of white noise at 85 dB, lasting 10 ms. The frequency of the sound pulses within a train was 40 Hz (i.e., one sound pulse every 25 ms). The train duration was 2 s, and the inter-train interval (from train onset to train onset) was 10 s. Inter-trial coherence (ITC) was calculated in the 35–45 Hz range on the separate trials, using a MATLAB algorithm. It measured the degree of coherence between individual trials: an ITC value of 0 indicates a complete absence of consistency, whereas an ITC value of 1 indicates perfect consistency. Statistical analysis of the ITC used Student’s *t*-test to compare the results from the two genotypes within each structure.

We qualitatively assessed whether there were differences among animals and between groups in the ability of mice to hear. After the ASSR recording session, animals were individually isolated in a quiet room, and when stationary and facing away from the investigator, a ring tone on an iPhone was played, and the startle response was noted.

### 2.4 Immunohistochemistry

As described in the Results section, while the P301L mice had an EEG phenotype distinct from WT mice, this phenotype was not progressive with age, contrary to our initial hypothesis. Thus, we performed a qualitative analysis of tau deposition to confirm that these indices of neurodegeneration were progressive in the mice used in our studies, as previously reported in detail by others (Lewis et al., 2000). Based on our previous experience in the analysis of neurodegeneration and neuroinflammation markers in Alzheimer’s pathology mouse models, we were confident that an *n* = 3 per group would be sufficient to qualitatively confirm these previous findings. P301L and WT mice, aged 3-, 6.5-, and 11-month-old mice (*n* = 3 per age and genotype), were anesthetized with isoflurane and perfused with saline. The brains were hemisected and fixed in 4% paraformaldehyde (PFA) for 24 h before being transferred to 30% sucrose for 3 days. The brains were embedded in optimal cutting temperature (OCT) compound and frozen at −80 °C. Sagittal sections (30 μm) were obtained using a cryostat (CM1510S; Leica Biosystems, Wetzlar, Germany). Primary antibodies used: mouse anti-AT8 (1:500, MN1020, Invitrogen, Carlsbad, CA), rabbit anti-CD11c (1:100, 97585S, Cell Signaling Technology, Danvers, MA, USA), rat anti-MHC-II (1:500, 107,601, Biolegend, San Diego, CA, USA), and rabbit anti-TREM2 conjugated to Alexa Fluor 647 (1:100, ab307858, Abcam, Cambridge, UK). The sections were blocked in PBS containing 5% goat serum and 0.3% Triton for 1 h at room temperature. Sections were incubated overnight at 4 °C with primary antibodies in PBS containing 1% bovine serum albumin (BSA) and 0.3% Triton. The following day, the sections were incubated for 1 h at room temperature with the appropriate secondary antibodies in PBS with 1% BSA and 0.3% Triton. The secondary antibodies used were goat anti-mouse Alexa Fluor 488 (1:1000, A11001, Thermo Fisher Scientific, Waltham, MA), goat anti-rabbit Alexa Fluor 488 (1:400, 111–547-003, Jackson ImmunoResearch Inc., West Grove, PA), and goat anti-rat Alexa Fluor 568 (1:1000, A11077, Thermo Fisher Scientific, Waltham, MA). Finally, TrueBlack (23007, Biotium, Fremont, CA, USA) was applied for 30 s to quench lipofuscin autofluorescence. Whole slides were imaged using an Olympus VS200 Slide Scanner microscope to capture fluorescent signals (Olympus Corporation, Waltham, MA, USA).

For the assessment of the abundance of different immune cell phenotypes, two sagittal sections were taken from each of the three mice per age and genotype and co-stained for CD11c, TREM2, and MHC-II. Sections ranged from 0.36–2.04 mm from the midline, with the majority of sections falling between 0.84–1.32 mm. Images were loaded into QuPath v0.5.1. Regions of interest were drawn around the brain regions with the highest immune activity (based on the 6.5- and 11-month-old P301L groups), which encompassed the inferior colliculus to the medulla oblongata, excluding the cerebellum. The inferior colliculus was chosen as the boundary mainly for ease of separation from the more rostral regions. Cell detection was performed via DAPI staining using the following parameters: background radius = 15 mm, median filter radius = 0 mm, sigma = 2 mm, minimum area = 10 mm^2^, maximum area = 80 mm^2^, threshold = 1,200, and cell expansion = 3 mm. QuPath machine learning object classifiers were then trained to identify cells positive for the 3 markers. Two separate object classifiers were trained to identify cells positive for CD11c and for TREM2 in regions of interest of area 800 × 800 mm, encompassing the variation in background noise and blood vessel staining seen across mice and brain sections. Classifiers were run sequentially, with the consequence that cells labeled for either marker or both markers were captured. Manual curation was performed afterwards to correct any obvious classification mistakes. Owing to the rarity of MHC-II cells and the difficulty of the classifier to correctly detect such cells against a background of blood vessel staining, MHC-II-positive cells were identified manually. Counts were aggregated by genotype and age and then normalized by dividing by the total number of DAPI-positive cells detected. Values are expressed as the number of cells positive for immune markers per 100,000 DAPI-positive nuclei. For statistical analysis, data were analyzed by 2-way ANOVA with factors of age and genotype (Prism 11.0.0, GraphPad Software, Boston, MA).

## 3 Results

In JNPL3(P301L) mice, htau^P301L^ expression is driven by the prion promoter and is largely neuronal (Lewis et al., 2000). Our focus was on investigating functional electrophysiological changes in these mice caused by the low level of htau^P301L^ expression in forebrain neurons. To put these functional electrophysiological changes into the perspective of pathological changes caused by htau^P301L^ expression, we qualitatively confirmed the age- and spatial-progressive accumulation in the forebrain of markers of tau neurofibrillary pathology (AT8 immunostaining) reported by others (Lewis et al., 2000; Sahara et al., 2002; Lin et al., 2003). We also showed that markers of neuroinflammation increase in parallel with the increase in AT8 immunostaining. As previously reported Lewis et al. (2000), P301L mice developed a hunched posture and ataxia at around 11 months of age that required study termination; consequently, our study used mice spanning 3 to 11 months of age. Note also that while the original characterization of P301L mice by the Lewis lab noted tau pathology in the cerebellum, we did not investigate cerebellar pathology in detail in our study, given that the focus was on functional changes in the forebrain.

### 3.1 Electrophysiological analyses

**Figure 1:**
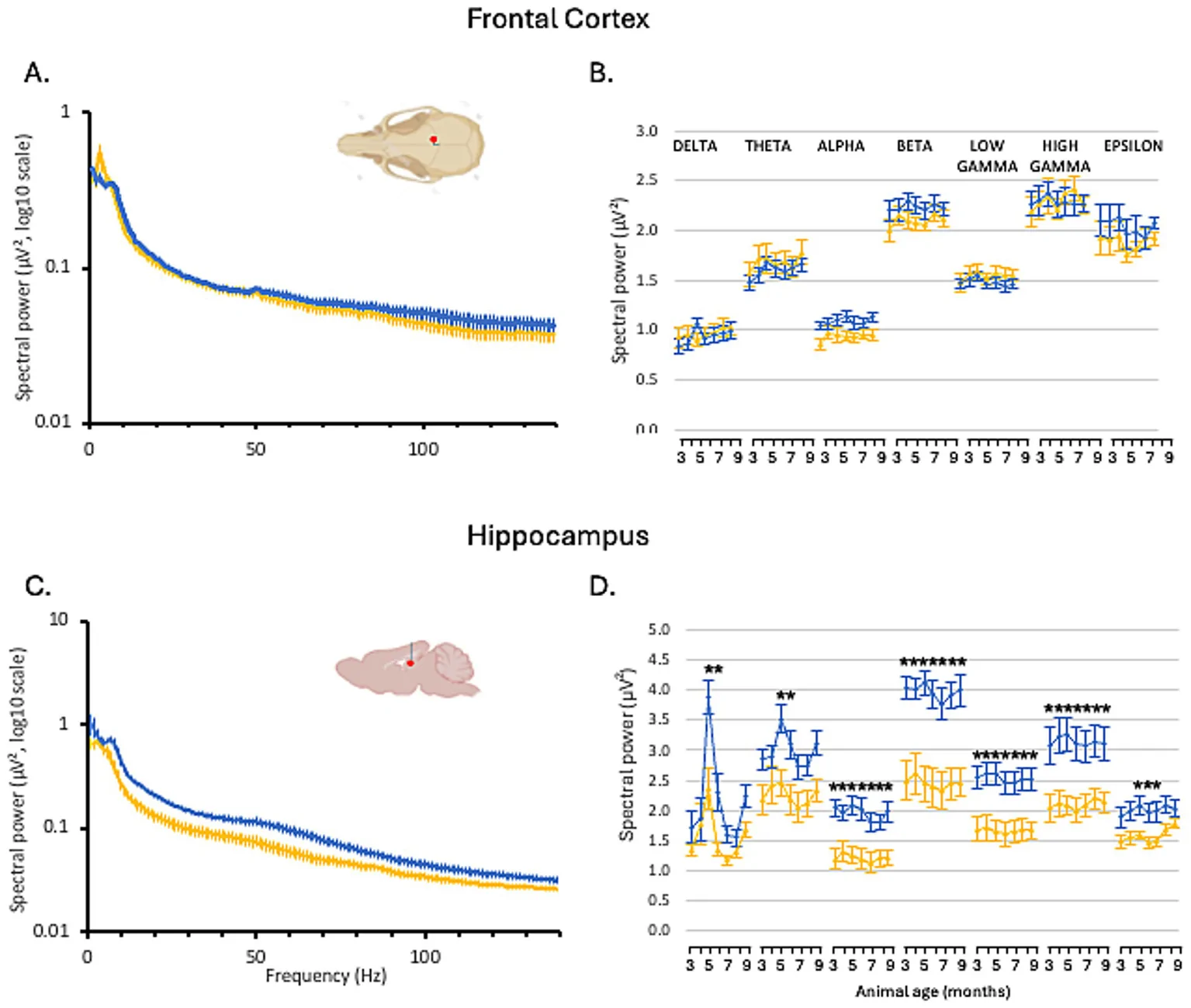
Spectral power of electrophysiological signals recorded at the cortical and hippocampal electrodes in P301L and WT mice. P301L and WT mice (*n* = 8 mice/genotype) were implanted with a surface electrode over the right frontal cortex and a depth electrode in the hippocampus. EEG was recorded in freely moving mice once per month from 3 to 9 months of age. **(A)** Mean (± S.E.M.) spectral power in the frontal cortex across all frequencies for P301L (blue) and WT (yellow) mice at 3 months of age. *Inset:* approximate placement of the right frontal screw electrode on the skull. **(B)** Mean (± S.E.M.) spectral power in the frontal cortex for each of the standard frequency bands for P301L or WT mice, where each point represents the average at each monthly recording, beginning at age 3 months and ending at age 9 months. **(C)** Mean (± S.E.M.) spectral power in the hippocampus across all frequencies for P301L and WT mice at 3 months of age. *Inset:* approximate placement of the hippocampal depth electrode in the sagittal view. **(D)** Mean (± S.E.M.) spectral power in the frontal cortex for each of the standard frequency bands for P301L or WT mice. Data for each frequency band **(B,D)** were analyzed using two-way ANOVA with genotype as a factor and time as a repeated measure (significance level *p* < 0.05). The results of the ANOVAs are shown in Table 1. For the frontal cortex data **(B)**, there were no statistically significant main effects of age or genotype in any frequency band at any age. For the hippocampal data **(D)**, there were statistically significant main effects of age and genotype, and further post-hoc analysis used the Dunnett test (significance level *p* < 0.05). * indicates a significant difference in EEG power between P301L and WT at the indicated ages.

**Figure 2:**
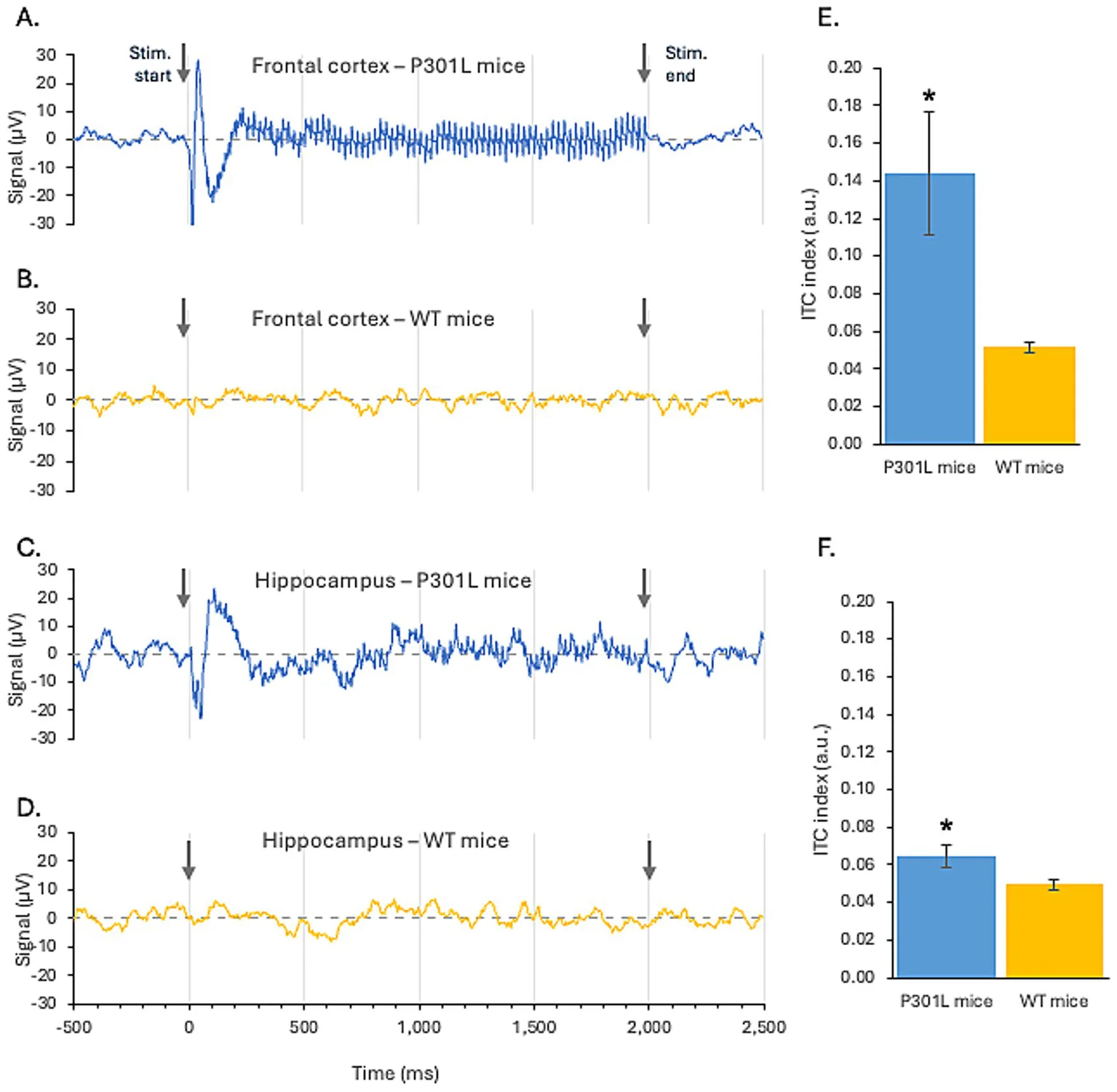
ASSR and ITC in P301L and WT mice were detected at the frontal cortical and hippocampal electrodes. ASSR at 40 Hz was investigated in P301L and WT mice at 10 months of age (*n* = 8 per genotype). Grand average ASSR in the right frontal cortex of P301L (**A**, blue) and WT mice (**B**, yellow) and the hippocampus of P301L mice **(C)** and WT mice **(D)**. For each mouse, train stimulation starting at 0 ms and ending at 2000 ms was averaged across 360 trains within the recording session. Data are represented as the mean ± S.E.M. Inter-trial coherence (ITC) in the right frontal cortex **(E)** and hippocampus **(F)** from P301L (blue) and WT (yellow) mice at 10 months of age. An ITC value of 0 indicates a complete absence of consistency, whereas an ITC value of 1 indicates perfect consistency. ITC was calculated for each mouse and averaged across groups (mean ± S.E.M., *n* = 8 per genotype). The index was calculated in the 35–45 Hz range, over the second half of the stimulation (1 to 2 s after the stimulation start). Asterisks (*) indicate statistically significant differences between P301L and WT mice using Student’s *t*-test (frontal cortex: *t*-test = 2.799, *df* = 14, *p* = 0.0142; hippocampus: *t*-test = 2.162, *df* = 14, *p* = 0.0484).

To investigate whether expression of htau^P301L^ affected neuronal function, we compared qEEG in P301L and WT mice from 3 to 9 months of age (Figure 1). Recordings were derived from screw electrodes implanted bilaterally over the frontal cortex and unilaterally over the parietal cortex and a depth electrode implanted in the hippocampus. To investigate higher-order neuronal integration, we also examined the entrainment of brain rhythmicity to a 40 Hz auditory stimulus (auditory steady-state response, ASSR) when the animals were 10 months of age (Figure 2).

#### 3.1.1 qEEG

In EEG recorded at electrodes over the frontal cortex, power spectral densities exhibited a standard shape, with high power in low frequencies and low power in high frequencies, as shown for recordings in 3-month-old mice in Figure 1A. Recordings from months 4 through 9 are presented in Supplementary Figure 1. In P301L mice, there was a subtle shift to greater power in higher frequencies compared to WT mice. A peak in power at 4 Hz in WT mice was shifted toward 7 Hz in P301L mice, and the power in frequencies from ~50 Hz through 140 Hz was slightly higher in P301L mice than in WT mice. However, there was no statistically significant difference between P301L and WT mice when spectra were divided into classical frequency bands, and total power in each band was computed and averaged over the 120-min recording sessions (Figure 1B; Table 1). Notably, there were no changes in power in any frequency band as a function of age in either P301L or WT mice. This pattern was the same for the EEG recorded at the electrode over the parietal cortex (not shown).

**Table 1:** Statistical analyses of changes in the spectral power of electrophysiological signatures at cortical and hippocampal electrodes in P301L and WT mice.

Spectral band	Age	Genotype	Age × genotype
Right frontal cortex			
Delta 1–4 Hz	F(6, 84) = 1.634 NS	F(1, 14) = 0.05089 NS	F(6, 84) = 1.313 NS
Theta 4–8 Hz	F(6, 84) = 1.714 NS	F(1, 14) = 0.5702 NS	F(6, 84) = 0.3172 NS
Alpha 8–12 Hz	F(6, 84) = 1.431 NS	F(1, 14) = 6.429 *p* = 0.0238	F(6, 84) = 0.7016 NS
Beta 12–30 Hz	F(6, 84) = 1.004 NS	F(1, 14) = 2.235 NS	F(6, 84) = 0.4264 NS
Low Gamma 30–50 Hz	F(6, 84) = 1.001 NS	F(1, 14) = 1.018 NS	F(6, 84) = 0.3705 NS
High Gamma 50–90 Hz	F(6, 84) = 0.7377 NS	F(1, 14) = 0.0009561 NS	F(6, 84) = 0.5488 NS
Epsilon 90–140 Hz	F(6, 84) = 1.106 NS	F(1, 14) = 1.199 NS	F(6, 84) = 0.2992 NS
Hippocampus			
Delta 1–4 Hz	F(6, 84) = 18.06 *p* < 0.0001	F(1, 14) = 8.666 *p* = 0.0107	F(6, 84) = 3.111 *p* = 0.0084
Theta 4–8 Hz	F(6, 84) = 2.565 *p* = 0.0249	F(1, 14) = 12.25 *p* = 0.0035	F(6, 84) = 0.6228 NS
Alpha 8–12 Hz	F(6, 84) = 0.9952 NS	F(1, 14) = 19.71 *p* = 0.0006	F(6, 84) = 0.5032 NS
Beta 12–30 Hz	F(6, 84) = 0.4890 NS	F(1, 14) = 27.67 *p* = 0.0001	F(6, 84) = 0.1287 NS
Low Gamma 30–50 Hz	F(6, 84) = 0.3837 NS	F(1, 14) = 14.26 *p* = 0.0020	F(6, 84) = 0.08832 NS
High Gamma 50–90 Hz	F(6, 84) = 0.5148 NS	F(1, 14) = 11.41 *p* = 0.0045	F(6, 84) = 0.3297 NS
Epsilon 90–140 Hz	F(6, 84) = 5.236 *p* = 0.0001	F(1, 14) = 6.851 *p* = 0.0203	F(6, 84) = 1.232 NS

Data from Figure 1. For each spectral band, power was analyzed using two-way ANOVA with genotype factors and repeated measures for age (significance level *p* < 0.05). Upper panel, Right frontal cortex; lower panel, Hippocampus.

The shift in power to higher frequencies in P301L mice was more pronounced in LFPs recorded with the hippocampal depth electrode (Figure 1C). The increase in power in P301L mice was evident across the frequency spectrum. These increases in power in P301L mice were present in the first recording at 3 months of age, and the increase was maintained across recording sessions through 9 months of age (Figure 1D). When spectral power was averaged over the 120-min recording sessions, the increases in the P301L mice reached statistical significance for the alpha, beta, and gamma bands. In the 2-way ANOVAs for each of these spectral bands, there was a significant main effect of genotype, but no main effect of age and no genotype × age interactions (Table 1). Post-hoc analyses indicated that the increases in power in the P301L mice were statistically significant at each age (Figure 1D). For the delta, theta, and epsilon bands, there were statistically significant main effects for genotype and age (Table 1). However, there was a statistically significant genotype × age interaction only for the delta band. *Post-hoc* analysis indicated that the increases in power for the P301L mice in the delta and theta bands reached statistical significance only at 5 and 6 months of age and for the epsilon bands at 5, 6, and 7 months of age (Figure 1D). Nonetheless, it is clear from the data in Figure 1 that there were no systematic age-related changes in power in either P301L or WT mice with increasing age.

**Figure 3:**
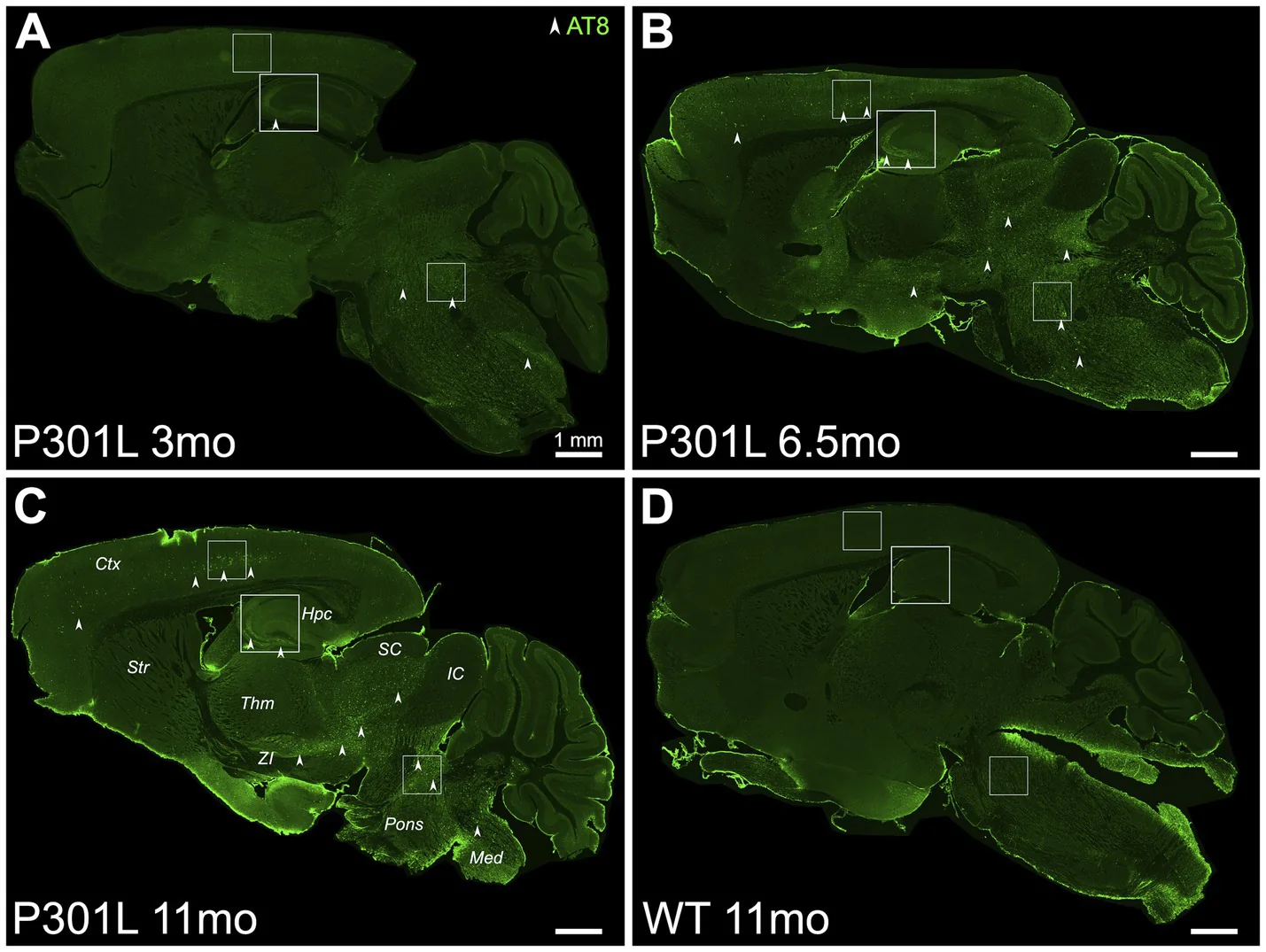
AT8 immunoreactivity in sagittal sections of brain from P301L and WT mice. Mouse brains were fixed, and sagittal sections (30 μm) approximately 1.2–2 mm from midline were stained for AT8 immunoreactivity (*n* = 3 mice per genotype and age). Sections were scanned at 20x magnification. AT8 immunoreactivity appears as bright green puncta and cell bodies. Representative images are shown; all images have the same brightness/contrast. Arrowheads indicate the areas of immunoreactivity. Boxes highlight the areas shown at greater magnification in Figure 4. Scale bar 1 mm. **(A)** 3-month-old P301L; **(B)** 6.5-month-old P301L; **(C)** 11-month-old P301L; **(D)** 11-month-old WT. Brain regions are labeled on **(C)**: Ctx, Cortex; Str, Striatum; Hpc, Hippocampus; Thm, Thalamus; ZI, Zona incerta; SC, Superior colliculus; IC, Inferior colliculus; Med, Medulla oblongata.

#### 3.1.2 ASSR

Specific oscillations evoked by sensory stimulations, such as the ASSR, represent the coordinated function of neuronal ensembles at different levels of organization and, as such, are studied to gain insight into deeper levels of neuronal information processing (Sivarao, 2015; Brenner et al., 2009). The ability of a 40 Hz tone to entrain the EEG (ASSR) was assessed at the 10-month time point. Entrainment to the 40 Hz tone was detected at the electrodes over the frontal cortex in five of the eight P301L mice (Figure 2A). Entrainment was characterized as a prominent initial response followed by a visible oscillation locked to the 40 Hz stimulations during the latter part of the train. In contrast, none of the WT mice showed prominent entrainment at any of their cortical electrodes. Consequently, the average EEG signal from these eight mice exhibited a flat profile during the 40 Hz stimulus (Figure 2B). Entrainment was also detected at the hippocampal electrode in the P301L mice, although the response was more variable and less clear visually (Figure 2C). Again, none of the WT mice showed prominent entrainment at the hippocampal electrodes (Figure 2D).

To further investigate the difference in ASSR between P301L and WT mice, inter-trial coherence (ITC) was calculated for each mouse over the second half of the stimulation train and averaged across animals for each genotype. ITC indices were higher in P301L mice than in WT mice in both cortex and hippocampus. The group-average ITC index in the right frontal cortex was significantly higher in P301L mice than in WT mice (*p* = 0.0142, Figure 2E). While the difference in the hippocampus between the groups was smaller, it still reached statistical significance (*p* = 0.0484, Figure 2F). In WT mice, the ITC indices were relatively low in both the hippocampus and cortex, consistent with the lack of ASSR entrainment observed in these animals.

There was no difference between P301L and WT mice in the number of animals that demonstrated a startle response to an iPhone ringtone. Furthermore, there was no correlation among the P301L mice between ASSR responsiveness and the startle response. These observations suggest that the difference between the ASSR responders and non-responders was not due to gross changes in auditory function or the ability to hear the 40 Hz stimulus.

### 3.2 Immunohistochemical analyses

While there was an effect of P301L expression on electrophysiological measures, it was striking that these changes were not age-related. This is in contrast to the previously reported age-related increase in tau pathology (Lewis et al., 2000; Lin et al., 2003). Thus, we sought to confirm that age-related tau pathology developed in the P301L mice used in this study to aid in the interpretation of the electrophysiological results.

#### 3.2.1 AT8

**Figure 4:**
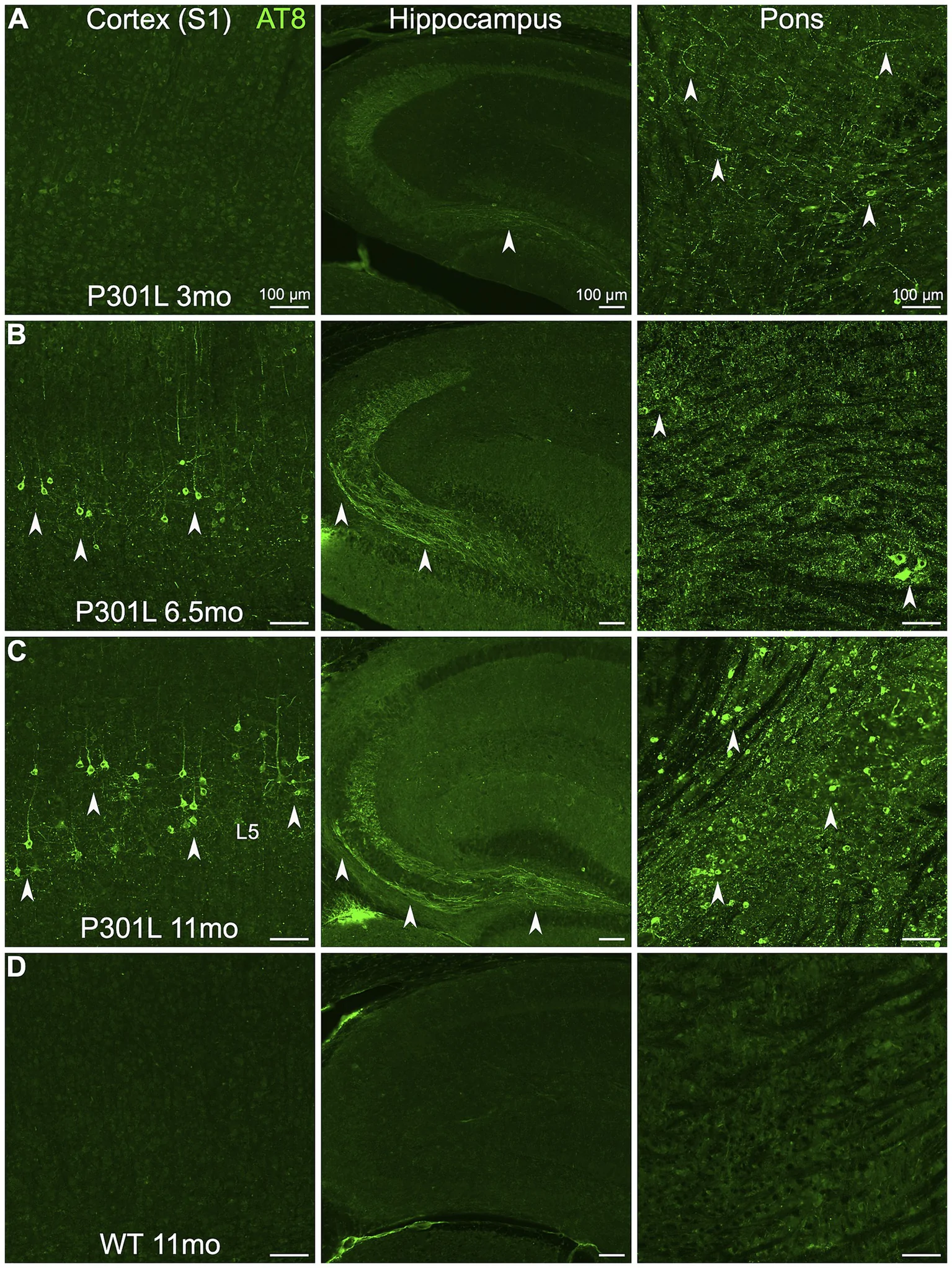
AT8 immunoreactivity in sagittal sections of the cortex, hippocampus, and pons of P301L and WT mice. Images are higher-magnification views of the sections shown in Figure 3. AT8 immunoreactivity appears as bright green puncta and larger cell bodies, highlighted by white arrowheads. Fields of view are—cortex: 800 μm × 800 μm; hippocampus: 1200 μm × 1,200 μm; pons: 800 μm × 800 μm. Left column: cortex (primary somatosensory/motor), middle column: hippocampus, right column: pons. Rows: **(A)** 3-month-old P301L; **(B)** 6.5-month-old P301L; **(C)** 11-month-old P301L; **(D)** 11-month-old WT.

The monoclonal antibody AT8 detects tau phosphorylated at Ser205 and Ser208 (Goedert et al., 1995). An increase in AT8 immunoreactivity is a consistent finding associated with neurofibrillary pathology in the AD brain and tauopathies (Augustinack et al., 2002). We observed AT8 immunoreactivity in P301L mice that developed with age from 3 to 11 months. AT8 staining was minimal in WT mice up to 11 months (Figures 3A–D, 4A–D).

At 3 months, AT8 immunoreactivity was most evident throughout the hindbrain (pons and medulla) (Figures 3A, 4A). Staining was punctate and appeared as chains, possibly following fibrous elements, such as neuronal axons. There were occasional AT8 immunoreactive cell bodies. There was also light AT8 immunoreactivity in the hippocampal pyramidal cell layer. In the hippocampus, AT8 staining was less punctate than in the hindbrain. There was little to no AT8 immunoreactivity in the cortex, thalamus, or striatum.

At 6.5 months of age, AT8 immunoreactivity was more intense in the hindbrain and hippocampus, and was also evident in the cortex, midbrain (superior and inferior colliculi), and zona incerta (Figures 3B, 4B). The punctate AT8 immunoreactivity in the hindbrain was more broadly distributed, and the fibrous patterning was less apparent, possibly obscured by the overall increase in density of puncta. There were more AT8-positive cell bodies in the hindbrain, and the intensity of AT8 immunoreactivity per cell appeared to be increased. There were also AT8 immunoreactive cell bodies in the cortex, with the highest density in deep layers in the region of the primary somatosensory and motor cortex (boxed area in cortex in Figure 3B), and had the shape of pyramidal neurons. In these neurons, AT8 immunoreactivity appeared to extend into the proximal apical dendrites (Figure 4B). AT8 immunoreactivity also increased in the hippocampus and appeared more intense in the CA3 region compared to the lighter but more uniform distribution observed at 3 months. The AT8 staining pattern in the hippocampus again appeared to lack the puncta observed in the hindbrain and cortex. There remained little to no AT8 immunoreactivity in the thalamus and striatum.

At 11 months of age, the patterns of AT8 immunoreactivity had increased further in intensity in the hindbrain, midbrain, and cortex compared to that at 6.5 months of age (Figures 3C, 4C). There was an increased number of AT8 immunoreactive cell bodies in the somatosensory/motor cortex. The AT8 immunoreactive cell bodies were more clearly of a pyramidal neuron shape, and immunoreactivity extended into both the proximal apical dendrites and basilar dendrites in some cases (Figure 4C). AT8 immunoreactive elements remained largely absent in the thalamus and striatum.

#### 3.2.2 Neuroinflammation markers

It is now widely appreciated that neuronal pathology is accompanied by neuroinflammation (Ransohoff, 2016; Adamu et al., 2024). One index of neuroinflammation is the presence of innate immune cells that are not found in the healthy brain but accumulate in areas of pathology. Such cells have been most widely studied in association with amyloid plaques in Alzheimer’s disease. One prominent population of such cells, now commonly referred to as ‘disease-associated microglia’ or DAMs, has several signature markers, including TREM2, CD11c, and MHC-II (Keren-Shaul et al., 2017). Immune cells expressing DAM markers have been reported in several disease states, including tauopathies (Deczkowska et al., 2018). Thus, we investigated the presence of TREM2, CD11c, and MHC-II immunoreactive cells as another index of the development of pathology associated with htau^P301L^ expression.

**Figure 5:**
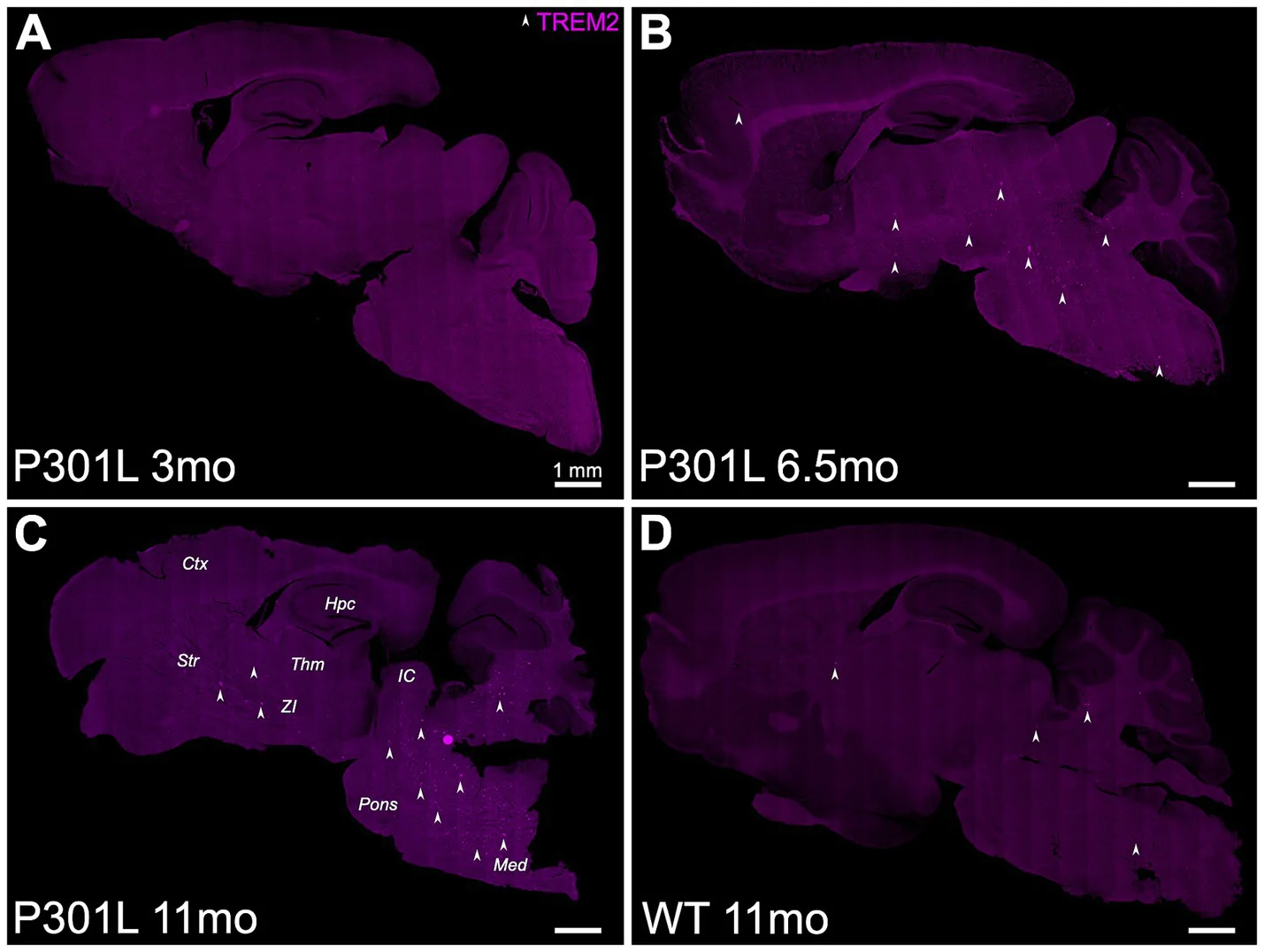
TREM2 immunoreactivity in sagittal sections of brain from P301L and WT mice. Mouse brains were fixed, and sagittal sections (30 μm) approximately 0.6–1.32 mm from midline were stained for TREM2 immunoreactivity (*n* = 3 mice per genotype and age). Sections were scanned at 20x magnification. TREM2 immunoreactive cells are bright magenta. Arrowheads highlight cell clusters. Scale bar 1 mm. **(A)** 3-month-old P301L; **(B)** 6.5-month-old P301L; **(C)** 11-month-old P301L; **(D)** 11-month-old WT. Brain regions are labeled on **(C)**: Ctx, Cortex; Str, Striatum; Hpc, Hippocampus; Thm, Thalamus; ZI, Zona Incerta; SC, Superior Colliculus; IC, Inferior Colliculus; Med, Medulla Oblongata.

We observed an accumulation of TREM2 immunoreactive cells in P301L mice (Figures 5A–D). The presence of these cells was absent at 3 months of age (Figure 5A), peaked in abundance at 6.5 months of age (Figure 5B), and remained elevated at 11 months of age (Figure 5C). TREM2 + cells were most apparent in the midbrain, the zona incerta, the hypothalamus, and the hindbrain. The presence of TREM2^+^ cells was variable throughout the rest of the brain. TREM2 immunoreactive cells were also present in WT mice at 11 months of age but were very rare compared to the P301L mice at this age (Figure 5D). Thus, the distribution of TREM2^+^ cells tended to follow the genotype, age, and spatial distribution patterns of AT8 immunoreactivity (Figures 3, 5).

**Figure 6:**
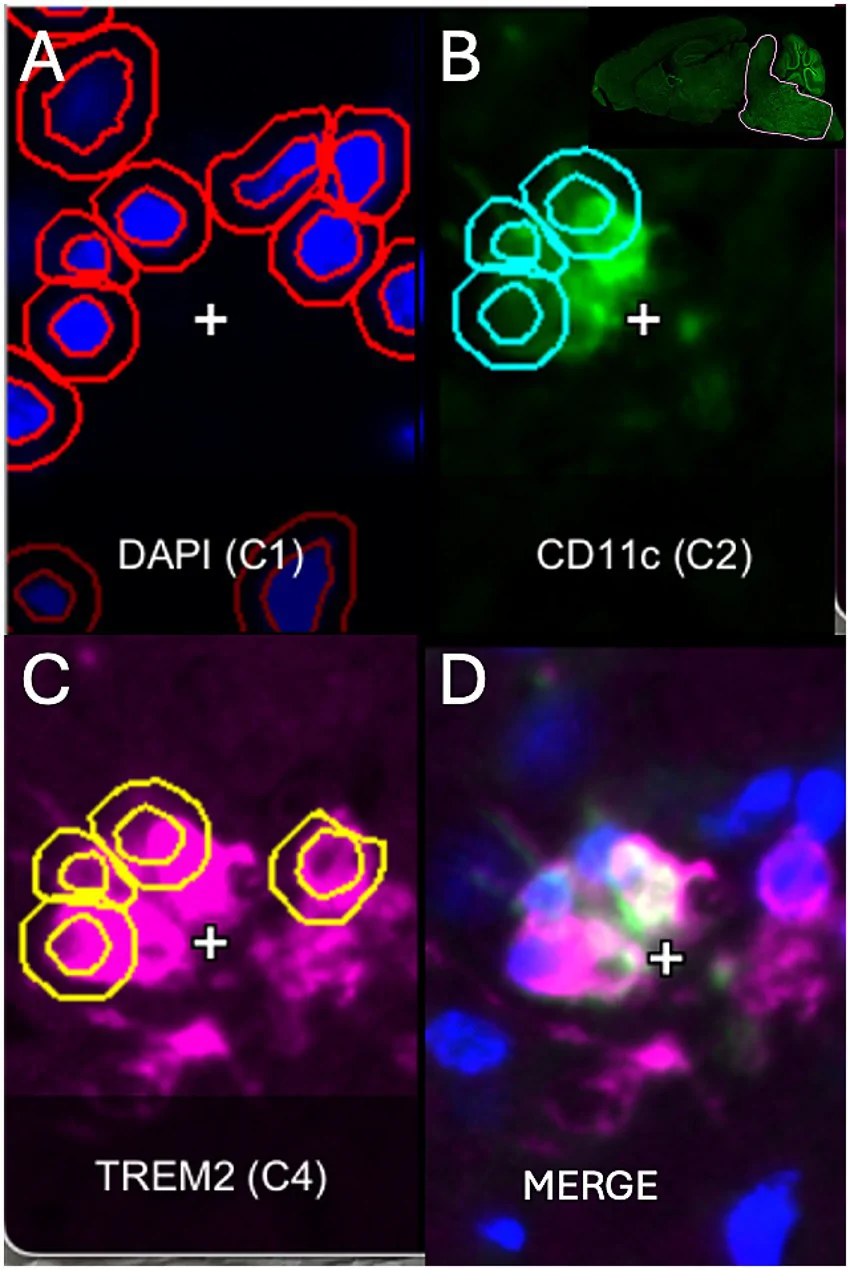
Quantitation of TREM2, CD11c, and MHC-II immunoreactive cells in the hindbrain of P301L and WT mice. Mouse brains were fixed, and sagittal sections (30 μm) were co-stained for CD11c, TREM2, and MHC-II immunoreactivity (*n* = 3 mice per genotype and age). The majority of sections were between 0.84 and 1.32 mm from midline. Two sections per mouse were scanned at 20x magnification and analyzed using QuPath v0.5.1. Regions of interest on the sagittal sections for cell counting were drawn from the inferior colliculus to the medulla oblongata, excluding the cerebellum (representative ROI, inset in **(B)**). Shown are representative 70 μm x 70 μm fields capturing single cells in an 11-month-old P301L mouse—**(A)** DAPI (blue nuclei, surrounded by red masks generated in QuPath for counting), **(B)** CD11c (green cells, light blue masks), **(C)** TREM2 (pink, yellow masks), Merged image **(D)**. MHC-II not shown. DAPI-stained cell nuclei were first identified, and then object classifiers were trained to identify cells positive for CD11c and TREM2. Cell counts are in Figure 7.

**Figure 7:**
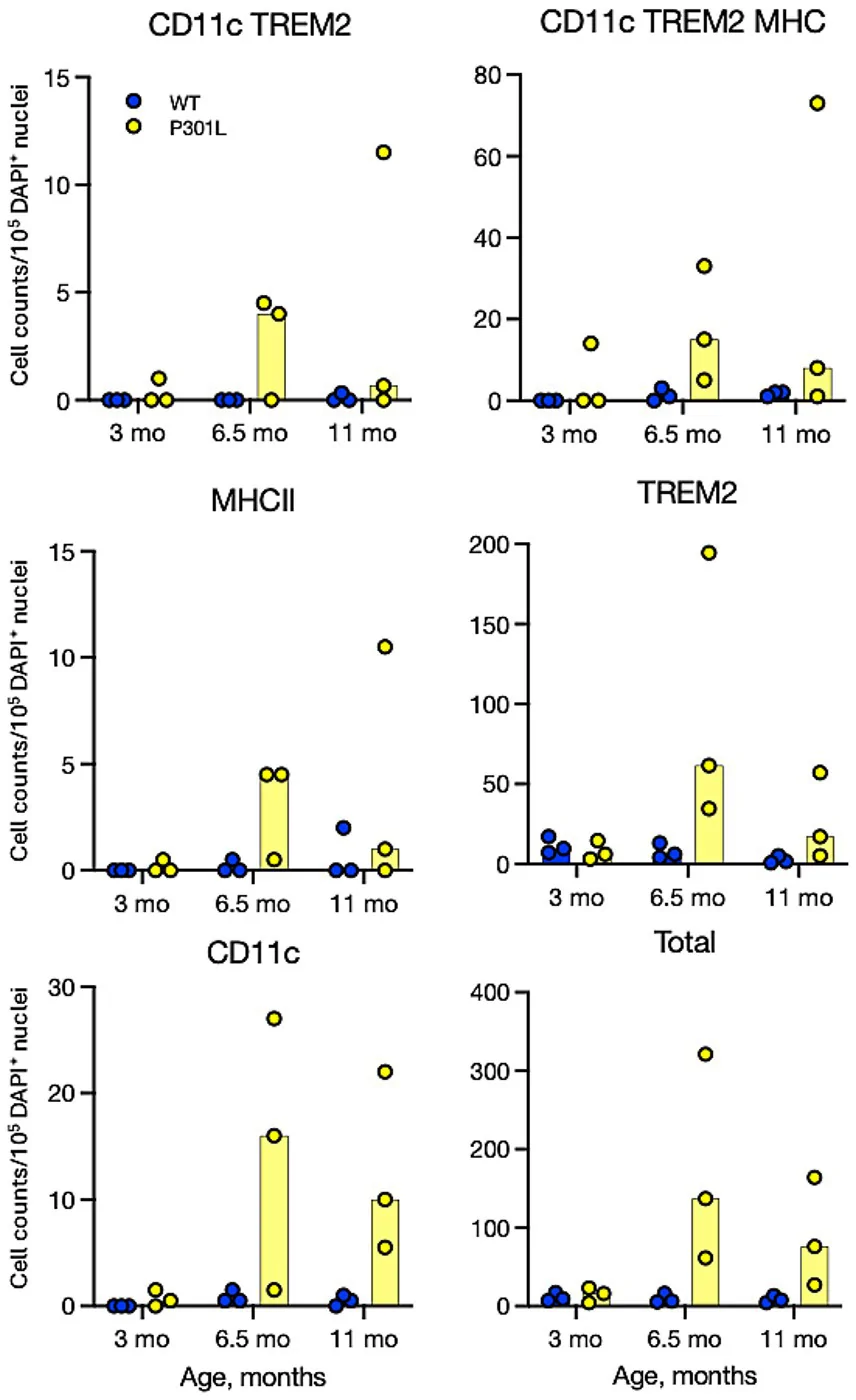
Normalized counts of cells positive for CD11c, MHC-II, and TREM2 in midbrain and hindbrain mouse brains were fixed, and sagittal sections (30 μm) were stained for CD11c, TREM2, and MHCII immunoreactivity (*n* = 3 mice per genotype and age; 2 brain sections per mouse). Cells strongly stained for each marker (Figure 6) were counted using a machine-learning object classifier in QuPath within a region of interest from the inferior colliculus to the medulla oblongata. Counts for each of the two sections per mouse were averaged. The average number of cells detected is expressed per 100,000 DAPI^+^ nuclei (values rounded to the nearest whole number). In each graph, the number of cells per mouse is shown as a dot (WT, yellow; P301L, blue). Bars are the group average. Total is the total number of cells positive for each of the markers (CD11c, MHCII, TREM2, CD11c/TREM2, and CD11c/TREM2/MHCII). Note that the Y axes (cell count) differ for each of the cell types counted for clarity.

To further characterize TREM2^+^ cells, we co-stained for the expression of CD11c and MHC-II and surveyed how cells with various combinations of these markers appear with age. We counted cells (Figure 6, representative images from a P301L mouse at 11 months of age) that were moderately to strongly immunoreactive for each of the markers in 3-, 6.5-, and 11-month-old animals, selecting the region of interest (ROI) from the inferior colliculus to the medulla oblongata, excluding the cerebellum (Figure 6B, inset). Immunoreactive cells were of highest abundance in the P310L mice at 6.5 and 11 months of age. The majority of cells immunoreactive for TREM2 (Figure 6A) were also immunoreactive for CD11c (Figure 6B). Less abundant were cells immunoreactive for TREM2 only or triple stained for TREM2, CD11c, and MHC-II, and there was also a small population of cells immunoreactive for MHC-II only. In WT mice, rare TREM2-immunoreactive cells did not co-stain with CD11c or MHC-II. Interestingly, these cells were the most abundant in 3-month-old animals. Rare TREM2^+^ and CD11c^+^ cells were observed in WT mice only at 11 months of age. Cell counts are presented in Figure 7. A 2-way ANOVA of total cell counts with factors of age and genotype returned a statistically significant difference in cell counts for genotype (F(1, 12) = 8.042; *p* = 0.015). There were trends for the effect of age and an age × genotype interaction, but these did not reach statistical significance.

## 4 Discussion

The tau proteins are exceptional in the degree to which dysfunction contributes to neurodegenerative disease (Stoothoff and Johnson, 2005; Spillantini and Goedert, 2013; Ballatore et al., 2007). Identifying the mechanisms of tau-related pathology is critical to understanding the diversity of these diseases and developing therapeutic interventions. In this study, we were interested in identifying EEG phenotypes that emerge with age-related tau deposition in JNPL3 (P301L) mice that may be used as outcomes in the testing of experimental therapeutics. We hypothesized that distinct EEG phenotypes would emerge progressively with age in P310L mice in temporal parallel with the emergence of tau deposition, as described by others (Lewis et al., 2000; Sahara et al., 2002; Lin et al., 2003). While we found evidence for altered neuronal excitability in these mice, these changes were not correlated with the age-related time course for tau deposition and indices of neuroinflammation, which are putative correlates of neurodegeneration. Instead, we hypothesize that altered neuronal activity in the P301L mice was due to htau^P301L^ expression causing dysregulation of physiological tau functions.

The neuronal oscillations embedded in the EEG represent the synchronized communication of nested neuronal ensembles carried at different oscillatory frequencies. Power in the classical EEG frequency bands reflects ensembles that are engaged and the information carried among the ensembles (Buzsaki and Draguhn, 2004). Specific oscillations evoked by sensory stimulations, such as the ASSR, represent the coordinated function of neuronal ensembles at different levels of organization and as such are studied to gain insight into deeper levels of neuronal information processing (Sivarao, 2015; Brenner et al., 2009). Differences in power within and/or across EEG frequency bands in the absence of stimulation or in oscillations evoked by sensory input, such as in the ASSR paradigm, may serve as functional indices of neurodegenerative processes. In this study, differences between the P301L and WT mice were observed. EEG analyses similar to those conducted here are also used to probe brain pathologies in human disease, including Alzheimer’s disease and other tauopathies (Babiloni et al., 2024; Cope et al., 2022; Cassani et al., 2018).

While we saw no age-related changes in the EEG in P301L mice, we did confirm age-related changes similar to those reported in previous characterizations of these mice (Lewis et al., 2000). This included the development of ataxia and a hunched posture with age, which required euthanasia at approximately 11 months. More germane to the EEG measurements, we confirmed that there was an age-related deposition of AT8-positive tau aggregates (Goedert et al., 1995). We also report an initial qualitative characterization of the immune response to the developing tau pathology in P301L mice. The accumulation of AT8 immunoreactivity paralleled the accumulation of TREM2^+^ immune cells. The TREM2^+^ immune cells were also typically positive for the expression of CD11c and, less frequently, positive for expression of MHC-II. These 3 markers are signatures for innate immune cells that are absent from the healthy brain but accumulate in response to brain injury or disease (Deczkowska et al., 2018). Such cells were described as ‘disease-associated microglia’ in an APP/PS1 amyloid-depositing mouse model (Keren-Shaul et al., 2017), and similar cells have been described in other amyloid-depositing mice, as well as in mouse models of brain injury and neurodegenerative pathology (Deczkowska et al., 2018; Salter and Stevens, 2017; Butovsky and Weiner, 2018). This pattern of responses may be characterized as a neuroinflammatory response associated with tau deposition, putatively another confirmatory manifestation of neurodegeneration.

The fact that the EEG phenotype in P301L mice did not change with age, and the apparent lack of robust neurodegeneration in frontal brain regions, strongly suggests that the EEG phenotype is functional, that is, it is the result of htau^P301L^ expression disrupting physiological tau function rather than neurodegeneration. We characterize the phenotype as an increase in neuronal excitability based on the observation that hippocampal LFP power was elevated, and the spectrum was shifted toward power in the higher frequency bands. The increase in excitability is also inferred from the finding that an ASSR was more readily induced in the P301L mice compared to the WT control mice. It is notable that the difference in ASSR was most strongly detected at frontal electrodes, where increases in EEG power spectrum were modest, further supporting the conclusion that tau-induced effects were functional rather than due to neurodegeneration.

The fact that htau^P301L^ expression may affect neuronal function and cause changes in electrophysiological signatures that are not directly related to pathological neurodegeneration is consistent with the physiological role of tau in the regulation of microtubule dynamics in axons (Wang and Mandelkow, 2016; Arendt et al., 2016; Parra Bravo et al., 2024). There are several reports of a similar dissociation in other tauopathy mouse models. In a P301L mouse line developed by Terwel et al. (2005), Boekhoorn et al. found an increase in sensitivity to the induction of hippocampal long-term potentiation that preceded the development of neurodegenerative changes (Boekhoorn et al., 2006). In the Tg4510 mouse line, where high levels of tau P301L are inducibly expressed, electrophysiological changes in frontal cortical pyramidal neurons did not correlate with progressive neuronal dystrophy (Crimins et al., 2012). Morgan et al. (2008) in the JNPL3 mice and Boekhoorn et al. (2006) in the Terwel P301L mouse model reported increases in performance in cognitive tasks relative to WT mice, which is consistent with a change in neuronal function and counterintuitive that this would result from neurodegeneration. Furthermore, Santacruz et al. (2005) reported that suppression of tau expression rescued functional behavioral deficits in a tau-overexpressing mouse line despite neurofibrillary pathology continuing to develop. However, a more common finding is the correlation between the age-dependent development of tau pathology and neuronal hypofunction phenotypes, as summarized in a meta-analysis of more than 20 preclinical studies by García-Carlos et al. (2025). Research into the role of tau in human neurodegenerative disease is primarily focused on how tau dysfunction progressively damages or kills neurons. In order to induce progressive neurodegeneration in mice, models have tended toward the complex and aggressive, featuring very high levels of tau expression, tau expression restricted to neuronal subpopulations instead of the broad expression that occurs naturally, and tau expression combined with other genetic manipulations that synergize in the development of neurodegeneration (Sahara and Yanai, 2023; Götz et al., 2007). Such aggressive manipulations and their removal from physiological tau disposition may mask functional aspects of tau pathophysiology, as detected in the JNPL3 mice in this study. In this context, it would be interesting to consider the characteristics of the JNPL3 P301L mice used in this study. In these mice, htau^P301L^ expression is constitutive and driven by the prion promoter, mimicking the expression of tau in diverse neuronal populations, and levels of htau^P301L^ are only 2-fold higher than levels of endogenous tau. Thus, the tau disposition in these mice is not far removed from the physiological condition. As was originally reported and as we also find here, frontal regions are relatively spared of neurodegeneration. This may have allowed for the observations here of the functional effects of tau expression on neuronal excitability in frontal brain regions.

A limitation of the present study is that it does not shed light on the degree to which the P301L substitution contributed to the EEG phenotype, rather than simply tau overexpression. A more ideal comparator for use with JNPL (P301L) mice would have been a line in which htau expression was driven by the prion promoter to the same levels as htau^P301L^. The question of the degree to which specific tau variants may contribute to functional phenotypes is significant to the human condition. The P301L mutation is associated with FTDP-17, a form of frontotemporal dementia with parkinsonism (Spillantini et al., 2000). However, over 100 mutations in tau have been identified, and the broad class of tauopathies is remarkable for variety in vulnerable neuronal populations, age of disease onset, and duration of disease progression (Langerscheidt et al., 2024). This includes AD, which may be considered a tauopathy in the absence of a genetically coded tau variant (Mi and Johnson, 2006). Thus, the degree to which tau variants are associated with distinct functional effects may help explain the diversity among this broad class of diseases, aside from the propensity of tau variants to form deposits. We cannot infer from the present data whether the increase in excitability observed in the EEG output was a direct result of a change in function in glutamatergic principal neurons or a secondary result of changes in network ensemble activity. Further research is needed to understand the underlying molecular mechanisms of the EEG phenotype in P301L mice.

The tauopathies feature prodromal phases of various lengths and manifestations before clinical diagnosis and detection of neurodegenerative changes (Benatar et al., 2024; Dubois et al., 2016). Notably, this includes an increase in the frequency of seizures and other indices of altered neuronal excitability (Sánchez et al., 2018; Targa Dias Anastacio et al., 2022; Samudra et al., 2023; Beagle et al., 2017; Kiernan and Petri, 2012). Given the physiological role of tau in regulating axonal microtubule dynamics, it is plausible that tau variation or dysregulation may cause functional changes that may contribute to increased neuronal excitability. Such alterations in function are putatively not benign in the long term and may provide the milieu for neurodegeneration, either as a result of tau deposition or by mechanisms that are no longer strictly tau dependent. It is a reasonable speculation that the hyperexcitability caused by aberrant tau expression detected in P301L mice may be related to these aspects of the human diseases. The lack of aggressive neurodegeneration in P301L mice allowed for the demonstration of this hyperexcitability as a functional phenomenon disambiguated from the development of neurodegeneration. Given that such prodromal changes may be a critical aspect of disease initiation and progression, further studies in less aggressive mouse models of tau dysregulation may uncover new therapeutic opportunities in ameliorating neuronal dysfunction beyond trying to stop established neurodegenerative processes, which has so far proved elusive.

## Data Availability

The raw data supporting the conclusions of this article will be made available by the authors, without undue reservation.

## Supplementary material

SUPPLEMENTARY FIGURE 1 Spectral power of the electrophysiological signals recorded at the cortical and hippocampal electrodes in P301L and WT mice. P301L and WT mice (*n* = 8 mice/genotype) were implanted with a surface electrode over right frontal cortex and a depth electrode in hippocampus. EEG was recorded in freely moving mice once per month from age 3 months to 9 months of age (see Methods). For each panel, mean (± S.E.M.) spectral power across all frequencies is displayed for P301L (blue) and WT (yellow) mice. Panels in the left column are for recordings from hippocampus and panels in the right column are from frontal cortex. Rows are recordings from each monthly recording from age 3 months to 9 months.

## References

[ref1] Adamu A. Li S. Gao F. Xue G. (2024). The role of neuroinflammation in neurodegenerative diseases: current understanding and future therapeutic targets. Front. Aging Neurosci. 16:1347987. doi: 10.3389/fnagi.2024.1347987, 38681666 PMC11045904

[ref2] Andreadis A. Brown W. M. Kosik K. S. (1992). Structure and novel exons of the human tau gene. Biochemistry 31, 10626–10633. doi: 10.1021/bi00158a027, 1420178

[ref3] Arendt T. Stieler J. T. Holzer M. (2016). Tau and tauopathies. Brain Res. Bull. 126, 238–292. doi: 10.1016/j.brainresbull.2016.08.018, 27615390

[ref4] Augustinack J. C. Schneider A. Mandelkow E.-M. Hyman B. T. (2002). Specific tau phosphorylation sites correlate with severity of neuronal cytopathology in Alzheimer's disease. Acta Neuropathol. 103, 26–35. doi: 10.1007/s004010100423, 11837744

[ref5] Babiloni C. Güntekin B. Yener G. Del Percio C.. qEEG methods to probe abnormal brain rhythms related to quiet vigilance in patients with dementia due to Alzheimer’s, Parkinson’s, and Lewy body diseases. In: Valeriani M Tommaso M de, editors. Psychophysiology Methods. New York, NY: Springer; (2024). 67–89.

[ref6] Ballatore C. Lee V. M.-Y. Trojanowski J. Q. (2007). Tau-mediated neurodegeneration in Alzheimer's disease and related disorders. Nat. Rev. Neurosci. 8, 663–672. doi: 10.1038/nrn2194, 17684513

[ref7] Beagle A. J. Darwish S. M. Ranasinghe K. G. La A. L. Karageorgiou E. Vossel K. A. (2017). Relative incidence of seizures and myoclonus in Alzheimer’s disease, dementia with Lewy bodies, and frontotemporal dementia. J. Alzheimer's Dis 60, 211–223. doi: 10.3233/JAD-170031, 28826176 PMC5608587

[ref8] Benatar M. Wuu J. Huey E. D. McMillan C. T. Petersen R. C. Postuma R. . (2024). The Miami framework for ALS and related neurodegenerative disorders: an integrated view of phenotype and biology. Nat. Rev. Neurol. 20, 364–376. doi: 10.1038/s41582-024-00961-z, 38769202 PMC11216694

[ref9] Benussi A. Alberici A. Samra K. Russell L. L. Greaves C. V. Bocchetta M. . (2022). Conceptual framework for the definition of preclinical and prodromal frontotemporal dementia. Alzheimers Dement. 18, 1408–1423. doi: 10.1002/alz.12485, 34874596

[ref10] Boekhoorn K. Terwel D. Biemans B. Borghgraef P. Wiegert O. Ramakers G. J. . (2006). Improved long-term potentiation and memory in young tau-P301L transgenic mice before onset of hyperphosphorylation and tauopathy. J. Neurosci. 26, 3514–3523. doi: 10.1523/jneurosci.5425-05.2006, 16571759 PMC6673867

[ref11] Borchelt D. R. Davis J. Fischer M. Lee M. K. Slunt H. H. Ratovitsky T. . (1996). A vector for expressing foreign genes in the brains and hearts of transgenic mice. Genet. Anal. 13, 159–163. doi: 10.1016/S1050-3862(96)00167-2, 9117892

[ref12] Braak H. Alafuzoff I. Arzberger T. Kretzschmar H. Tredici K. (2006). Staging of Alzheimer disease-associated neurofibrillary pathology using paraffin sections and immunocytochemistry. Acta Neuropathol. 112, 389–404. doi: 10.1007/s00401-006-0127-z, 16906426 PMC3906709

[ref13] Braak H. Braak E. (1995). Staging of Alzheimer's disease-related neurofibrillary changes. Neurobiol. Aging 16, 271–278. doi: 10.1016/0197-4580(95)00021-6, 7566337

[ref14] Brenner C. A. Krishnan G. P. Vohs J. L. Ahn W.-Y. Hetrick W. P. Morzorati S. L. . (2009). Steady state responses: electrophysiological assessment of sensory function in schizophrenia. Schizophr. Bull. 35, 1065–1077. doi: 10.1093/schbul/sbp091, 19726534 PMC2762626

[ref15] Butovsky O. Weiner H. L. (2018). Microglial signatures and their role in health and disease. Nat. Rev. Neurosci. 19, 622–635. doi: 10.1038/s41583-018-0057-5, 30206328 PMC7255106

[ref16] Buzsaki G. Draguhn A. (2004). Neuronal oscillations in cortical networks. Science 304, 1926–1929. 10.1126/science.1099745 15218136

[ref17] Cassani R. Estarellas M. San-Martin R. Fraga F. J. Falk T. H. (2018). Systematic review on resting-state EEG for Alzheimer’s disease diagnosis and progression assessment. Dis. Markers 2018, 1–26. doi: 10.1155/2018/5174815, 30405860 PMC6200063

[ref18] Citron M. Oltersdorf T. Haass C. McConlogue L. Hung A. Y. Seubert P. . (1992). Mutation of the beta-amyloid precursor protein in familial Alzheimer's disease increases beta-protein production. Nature 360, 672–674. doi: 10.1038/360672a0 1465129

[ref19] Cope Z. A. Murai T. Sukoff Rizzo S. J. (2022). Emerging electroencephalographic biomarkers to improve preclinical to clinical translation in Alzheimer’s disease. Front. Aging Neurosci. 14. doi: 10.3389/fnagi.2022.805063, 35250541 PMC8891809

[ref20] Crimins J. L. Rocher A. B. Luebke J. I. (2012). Electrophysiological changes precede morphological changes to frontal cortical pyramidal neurons in the rTg4510 mouse model of progressive tauopathy. Acta Neuropathol. 124, 777–795. doi: 10.1007/s00401-012-1038-9, 22976049 PMC3509230

[ref21] Deczkowska A. Keren-Shaul H. Weiner A. Colonna M. Schwartz M. Amit I. (2018). Disease-associated microglia: a universal immune sensor of neurodegeneration. Cell 173, 1073–1081. doi: 10.1016/j.cell.2018.05.003, 29775591

[ref22] Dubois B. Hampel H. Feldman H. H. Scheltens P. Aisen P. Andrieu S. . (2016). Preclinical Alzheimer's disease: definition, natural history, and diagnostic criteria. Alzheimers Dement. 12, 292–323. doi: 10.1016/j.jalz.2016.02.002, 27012484 PMC6417794

[ref23] García-Carlos C. A. Basurto-Islas G. Perry G. Campos-Ramírez C. Mondragón-Rodríguez S. (2025). Hyperphosphorylated tau induces cortical Hypoexcitability in transgenic mouse models: a meta-analysis. J. Integr. Neurosci. 24. doi: 10.31083/JIN39192, 40919630

[ref24] Goedert M. Jakes R. Vanmechelen E. (1995). Monoclonal antibody AT8 recognises tau protein phosphorylated AT both serine 202 and threonine 205. Neurosci. Lett. 189, 167–170. doi: 10.1016/0304-3940(95)11484-e 7624036

[ref25] Götz J. Deters N. Doldissen A. Bokhari L. Ke Y. Wiesner A. . (2007). A decade of tau transgenic animal models and beyond. Brain Pathol. 17, 91–103. doi: 10.1111/j.1750-3639.2007.00051.x, 17493043 PMC8095624

[ref26] Keren-Shaul H. Spinrad A. Weiner A. Matcovitch-Natan O. Dvir-Szternfeld R. Ulland T. K. . (2017). A unique microglia type associated with restricting development of Alzheimer's disease. Cell 169, 1276–1290.e17. doi: 10.1016/j.cell.2017.05.018, 28602351

[ref27] Kiernan M. C. Petri S. (2012). Hyperexcitability and Amyotrophic lateral Sclerosis. AAN Enterprises, 1544–1545. 10.1212/WNL.0b013e3182563c0a 22517105

[ref28] Kowall N. W. Kosik K. S. (1987). Axonal disruption and aberrant localization of tau protein characterize the neuropil pathology of Alzheimer's disease. Ann. Neurol. 22, 639–643. doi: 10.1002/ana.410220514, 3122646

[ref29] Langerscheidt F. Wied T. Al Kabbani M. A. van Eimeren T. Wunderlich G. Zempel H. (2024). Genetic forms of tauopathies: inherited causes and implications of Alzheimer's disease-like TAU pathology in primary and secondary tauopathies. J. Neurol. 271, 2992–3018. doi: 10.1007/s00415-024-12314-3, 38554150 PMC11136742

[ref30] Langness V. F. Simmons D. A. McHugh T. L. M. Butler I. R. R. Zhou J. Liu H. . (2025). Twenty years of therapeutic development in tauopathy mouse models: a scoping review. Alzheimers Dement. 21:e70578. doi: 10.1002/alz.70578, 40826256 PMC12360913

[ref31] Lee G. Cowan N. Kirschner M. (1988). The primary structure and heterogeneity of tau protein from mouse brain. Science 239, 285–288. doi: 10.1126/science.3122323, 3122323

[ref32] Lewis J. McGowan E. Rockwood J. Melrose H. Nacharaju P. Van Slegtenhorst M. . (2000). Neurofibrillary tangles, amyotrophy and progressive motor disturbance in mice expressing mutant (P301L) tau protein. Nat. Genet. 25, 402–405. doi: 10.1038/78078, 10932182

[ref33] Lin W.-L. Lewis J. Yen S.-H. Hutton M. Dickson D. W. (2003). Ultrastructural neuronal pathology in transgenic mice expressing mutant (P301L) human tau. J. Neurocytol. 32, 1091–1105. doi: 10.1023/B:NEUR.0000021904.61387.95, 15044841

[ref34] Mi K. Johnson G. V. (2006). The role of tau phosphorylation in the pathogenesis of Alzheimer's disease. Curr. Alzheimer Res. 3, 449–463. doi: 10.2174/156720506779025279, 17168644

[ref35] Morgan D. Munireddy S. Alamed J. DeLeon J. Diamond D. M. Bickford P. . (2008). Apparent behavioral benefits of tau overexpression in P301L tau transgenic mice. J. Alzheimer's Dis 15, 605–614. doi: 10.3233/JAD-2008-15407, 19096159 PMC3018352

[ref36] Mullan M. Crawford F. Axelman K. Houlden H. Lilius L. Winblad B. . (1992). A pathogenic mutation for probable Alzheimer's disease in the APP gene at the N-terminus of beta-amyloid. Nat. Genet. 1, 345–347. doi: 10.1038/ng0892-345 1302033

[ref37] Parra Bravo C. Naguib S. A. Gan L. (2024). Cellular and pathological functions of tau. Nat. Rev. Mol. Cell Biol. 25, 845–864. doi: 10.1038/s41580-024-00753-9, 39014245

[ref38] Paxinos G. Franklin K. B. (2019). Paxinos and Franklin's the Mouse brain in Stereotaxic Coordinates. Academic Press.

[ref39] Ransohoff R. M. (2016). How neuroinflammation contributes to neurodegeneration. Science 353, 777–783. doi: 10.1126/science.aag2590, 27540165

[ref40] Sahara N. Lewis J. DeTure M. McGowan E. Dickson D. W. Hutton M. . (2002). Assembly of tau in transgenic animals expressing P301L tau: alteration of phosphorylation and solubility. J. Neurochem. 83, 1498–1508. doi: 10.1046/j.1471-4159.2002.01241.x, 12472903

[ref41] Sahara N. Murayama M. Higuchi M. Tetsuya S. Takashima A. (2014). Biochemical distribution of tau protein in synaptosomal fraction of transgenic mice expressing human P301L tau. Front. Neurol. 5:2014. 10.3389/fneur.2014.00026 PMC3949102 24653715

[ref42] Sahara N. Yanai R. (2023). Limitations of human tau-expressing mouse models and novel approaches of mouse modeling for tauopathy. Front. Neurosci. 17. doi: 10.3389/fnins.2023.1149761, 37152607 PMC10157230

[ref43] Salter M. W. Stevens B. (2017). Microglia emerge as central players in brain disease. Nat. Med. 23, 1018–1027. doi: 10.1038/nm.4397, 28886007

[ref44] Samudra N. Ranasinghe K. Kirsch H. Rankin K. Miller B. (2023). Etiology and clinical significance of network hyperexcitability in alzheimer’s disease: unanswered questions and next steps. J. Alzheimer's Dis 92, 13–27. doi: 10.3233/JAD-220983, 36710680 PMC12990314

[ref45] Sánchez M. P. García-Cabrero A. M. Sánchez-Elexpuru G. Burgos D. F. Serratosa J. M. (2018). Tau-induced pathology in epilepsy and dementia: notions from patients and animal models. Int. J. Mol. Sci. 19:1092. doi: 10.3390/ijms19041092, 29621183 PMC5979593

[ref46] Santacruz K. Lewis J. Spires T. Paulson J. Kotilinek L. Ingelsson M. . (2005). Tau suppression in a neurodegenerative mouse model improves memory function. Science 309, 476–481. doi: 10.1126/science.1113694, 16020737 PMC1574647

[ref47] Serrats J. Vadodaria K. C. Brubaker W. Barker-Haliski M. White H. S. Evrard A. . (2025). ENX-101, a GABA_A_ receptor α2,3,5-selective positive allosteric modulator, displays antiseizure effects in rodent seizure and epilepsy models. Epilepsia 66, 2124–2136. doi: 10.1111/epi.18340, 40088186 PMC12169410

[ref48] Sivarao D. V. (2015). The 40-Hz auditory steady-state response: a selective biomarker for cortical NMDA function. Ann. N. Y. Acad. Sci. 1344, 27–36. doi: 10.1111/nyas.12739, 25809615

[ref49] Spillantini M. G. Goedert M. (2013). Tau pathology and neurodegeneration. Lancet Neurol. 12, 609–622. doi: 10.1016/S1474-4422(13)70090-5, 23684085

[ref50] Spillantini M. G. Van Swieten J. C. Goedert M. (2000). Tau gene mutations in frontotemporal dementia and parkinsonism linked to chromosome 17 (FTDP-17). Neurogenetics 2, 193–205. doi: 10.1007/s100489900084, 10983715

[ref51] Stoothoff W. H. Johnson G. V. W. (2005). Tau phosphorylation: physiological and pathological consequences. Biochim. Biophys. Acta (BBA) - Mol. Basis Dis. 1739, 280–297. doi: 10.1016/j.bbadis.2004.06.017, 15615646

[ref52] Targa Dias Anastacio H. Matosin N. Ooi L. (2022). Neuronal hyperexcitability in Alzheimer’s disease: what are the drivers behind this aberrant phenotype? Transl. Psychiatry 12:257. doi: 10.1038/s41398-022-02024-7, 35732622 PMC9217953

[ref53] Terwel D. Lasrado R. Snauwaert J. Vandeweert E. Van Haesendonck C. Borghgraef P. . (2005). Changed conformation of mutant tau-P301L underlies the moribund tauopathy, absent in progressive, nonlethal axonopathy of tau-4R/2N transgenic mice. J. Biol. Chem. 280, 3963–3973. doi: 10.1074/jbc.M409876200, 15509565

[ref54] Wang Y. Mandelkow E. (2016). Tau in physiology and pathology. Nat. Rev. Neurosci. 17, 22–35. doi: 10.1038/nrn.2015.1 26631930

[ref55] Zhang Y. Wu K.-M. Yang L. Dong Q. Yu J.-T. (2022). Tauopathies: new perspectives and challenges. Mol. Neurodegener. 17:28. doi: 10.1186/s13024-022-00533-z, 35392986 PMC8991707

